# Causal effect of fasting serum glucose on atherosclerotic cardiovascular disease: a multivariable Mendelian randomization

**DOI:** 10.4178/epih.e2024096

**Published:** 2024-12-06

**Authors:** Su Hyun Lee, Heejin Kimm, Byung-Wan Lee, Chung Mo Nam, So Young Kim, Sunmi Lee, Sun Ha Jee

**Affiliations:** 1 Department of Cancer Biomedical Science, Graduate School of Cancer Science and Policy, National Cancer Center, Goyang, Korea; 2 Department of Epidemiology and Health Promotion, Institute for Health Promotion, Graduate School of Public Health, Yonsei University, Seoul, Korea; 3 Department of Internal Medicine, Yonsei University College of Medicine, Seoul, Korea; 4 Department of Preventive Medicine, Yonsei University College of Medicine, Seoul, Korea; 5 Health Insurance Research Institute, National Health Insurance Service, Wonju, Korea

**Keywords:** Blood glucose, Cardiovascular disease, Mendelian randomization analysis

## Abstract

**OBJECTIVES:**

Observational studies have reported that diabetes is a risk factor that increases the risk of atherosclerotic cardiovascular disease (ASCVD). However, the causal relationship remains a matter of debate. This study aimed to analyze the relationship between fasting serum glucose (FSG) and ASCVD.

**METHODS:**

This study used data from the Korean Cancer Prevention Study-II (KCPS-II) Biobank, consisting of 159,844 people recruited with consent from 18 health examination centers from 2004 to 2013. Outcomes were confirmed based on diagnoses on hospital discharge summaries from National Health Insurance System. We used linear and non-linear Mendelian randomization (MR) methods. The outcome data were obtained from KCPS-II, and the exposure data were derived from the Korean Genome Epidemiology Study.

**RESULTS:**

First, a prospective cohort study estimated that for each 10 mg/dL increase in FSG level, the risk of ASCVD increased by 4% (hazard ratio [HR], 1.04; 95% confidence interval [CI], 1.03 to 1.05). Second, the 2-sample MR study showed that every 10 mg/dL increase in FSG influenced the risk of ASCVD (odds ratio [OR], 1.11; 95% CI, 1.04 to 1.18). Third, the multivariable MR study showed that the OR per 10 mg/dL increase in FSG on ASCVD was 1.14 (p<0.001). Similar results were found for a 10 mg/dL increase in FSG and ischemic heart disease (IHD), but a significant relationship with stroke was not found. When performing non-linear MR, a linear relationship was observed between fasting blood sugar and ASCVD, including IHD and stroke.

**CONCLUSIONS:**

FSG showed a linear and causal association with IHD, but not with stroke.

## GRAPHICAL ABSTRACT

[Fig f4-epih-46-e2024096]

## Key Message

Over the years, numerous epidemiological observational studies have reported that diabetes is a risk factor for cardiovascular and cerebrovascular diseases. However, most of these studies examining this association were conducted on Western populations, and no studies have been published using Korean data. This study employed a new methodology—multivariable Mendelian randomization—which is less affected by confounding factors and measurement errors, often highlighted as limitations of observational studies, to analyze the causal relationship between fasting glucose levels and cardiovascular and cerebrovascular diseases. The analysis revealed a causal relationship between fasting glucose levels and the risk of ischemic heart disease, while no causal association was observed with stroke.

## INTRODUCTION

Diabetes is a known risk factor that increases the risk of atherosclerotic cardiovascular disease (ASCVD). Previous meta-analyses and observational studies have supported the association between dysglycemia-related parameters, such as fasting glucose, hemoglobin A1c, and diabetes, with stroke [1] and cardiovascular disease (CVD) [2,3]. However, establishing causality has been challenging due to issues of confounding and reverse causality [4,5]. A primary source of bias in observational studies is the presence of unmeasured or inaccurately measured confounding variables. Additionally, uncertainties persist regarding the relationship between subthreshold blood glucose levels and CVD risks in the context of diabetes diagnosis, exemplified by the U-shaped relationship between blood glucose and CVD risk [6].

Gene-based Mendelian randomization (MR) is a recently developed method that addresses these issues by allowing for conclusions about causal associations under the assumption that genes are randomly assigned, thereby circumventing the influence of confounding variables [4,5].

To investigate whether fasting serum glucose (FSG) levels have a causal relationship with ASCVD risk in the Korean population, the objectives of this study were to analyze the relationship between FSG and CVD through an observational cohort study and to investigate the causal relationship between FSG and CVD using the MR method.

## MATERIALS AND METHODS

**Figure 1:**
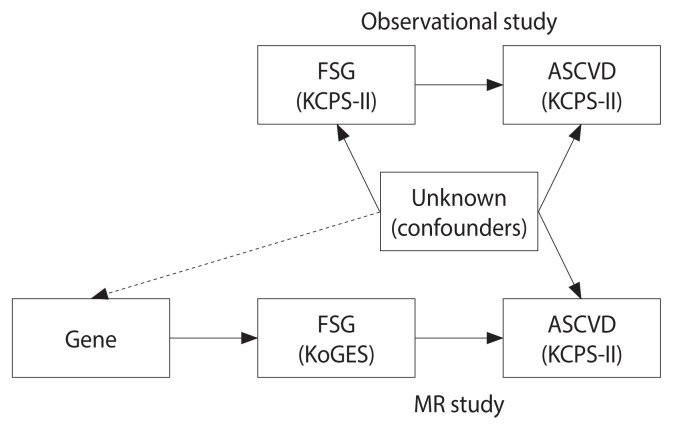
Research design of observational and Mendelian randomization (MR) studies. FSG, fasting serum glucose ; KCPS-II, Korean Cancer Prevention Study-II; ASCVD, atherosclerotic cardiovascular disease; KoGES, Korean Genome and Epidemiology Study.

The research designs of the observational and MR studies are shown in Figure 1. In observational studies, FSG is associated with confounding variables, which are accordingly controlled. In contrast, in MR research, genes are independent of confounding variables and are thus unaffected by them. The materials and methods used in the study are detailed below.

### Korean Cancer Prevention Study-II

The data used in this study were sourced from the Korean Cancer Prevention Study (KCPS)-II Biobank, which includes records from 159,844 individuals who visited 18 comprehensive examination centers between 2004 and 2013. These centers comprised 15 located in Seoul and Gyeonggi Province and 3 in other regions [7]. All participants provided informed consent. After excluding individuals with missing or extremely abnormal values for key variables, such as diabetes mellitus, those with a history of ASCVD, and those taking FSG-lowering medication, the study ultimately included 153,971 participants (Supplementary Material 1).

At baseline, all participants were asked to report their weight and height. Blood samples were collected after fasting for more than 8 hours, and levels of low-density lipoprotein (LDL)-cholesterol, high-density lipoprotein (HDL)-cholesterol, triglycerides (TG), and FSG were measured. FSG levels were determined using Hitachi-7600 analyzers (Hitachi, Tokyo, Japan).

We defined incident ASCVD as the first hospital admission for an ASCVD diagnosis (International Classification of Diseases, 10th revision [ICD-10] codes) using National Health Insurance Service (NHIS) data, or as death with ASCVD listed as the underlying cause if there was no relevant hospital admission (ICD-10 codes) [8]. The detailed outcomes of this study include ischemic heart disease (IHD; ICD-10 codes I20–I25), stroke (ICD-10 codes I60–I69), and other CVD (ICD-10 codes I10–I15, I44–I49, I50, I51, I70–I74, R96). IHD was categorized into two types: angina pectoris and acute myocardial infarction (AMI). Strokes were classified as either ischemic or hemorrhagic. A validation study conducted on the ICD codes for IHD and stroke among KCPS-II Biobank participants confirmed their validity at 93% and 83%, respectively [9–11].

### Korean Genome and Epidemiology Study

Korean Genome and Epidemiology Study (KoGES), a large-scale prospective genomic and epidemiological cohort study, offers long-term follow-up on common complex diseases and causes of death among Koreans. Genetic factors for FSG were identified in a total of 72,299 individuals. The average age of the participants was 54.65 years, and the mean FSG level was 93.72 mg/dL [12].

### Observational study

ASCVD participants with and without were compared in terms of socio-demographics, behaviors, past histories, and ASCVD risk factors. The associations between FSG and strokes, IHD, and AMI were examined separately. After confirming adherence to the proportionality assumption, we proceeded with the analysis using adjusted Cox proportional hazards models. Adjustments were made for baseline age, sex, HDL-cholesterol, LDL-cholesterol, systolic blood pressure (SBP), alcohol status, and smoking status.

### Genome-wide association study

To conduct MR, all subjects must have genetic test data. The quality control criteria applied for genome-wide association study (GWAS) analysis included a minor allele frequency of 0.01 and Hardy-Weinberg equilibrium less than 10^−6^. For the analysis, 6,804,815 single nucleotide polymorphisms (SNPs) were used for KCPS-II and 9,500,000 SNPs for KoGES.

To estimate the effect size of each SNP on FSG and cardiovascular events, logistic regression was employed, incorporating variables such as age, sex, chip type, and principal components. In this study, the GWAS analysis was conducted using PLINK 2.0.

### Two-sample Mendelian randomization

#### IVW method

The inverse-variance weighted (IVW) method, which utilizes multiple variants as instruments, is the most popular approach. An IVW meta-analysis of the ratio estimates from individual variants can be employed to determine the IVW estimate for uncorrelated variants [13]. Additionally, Cochran’s Q test and the MR-Egger intercept test will be conducted to assess heterogeneity in the instrumental variables [14].

#### MR-Egger

The MR-Egger method calculates the average pleiotropic effect using the intercept and determines the slope from the weighted regression of variant-outcome associations on variant-exposure associations. This approach allows for pleiotropic effects from any genetic variant, provided that these effects are independent of the variant-exposure associations, a stipulation known as the instrument strength independent of direct effect assumption [15].

#### Weighted median

Weighted median methods operate under the assumption that a greater number of variants accurately estimate the true causal effect compared to those estimating other quantities, provided that fewer than half of the variants are invalid instruments (the majority validity assumption) [16]. This approach is robust against outliers and is sensitive to the addition or removal of instrumental variables.

### Non-linear Mendelian randomization

We aimed to evaluate the shape of the association between exposure and outcome at the individual level by employing two methods: the fractional polynomial method and the piecewise linear method. By conditioning on quantities of instrumental variable–free exposure, we estimated the localized average causal effect [17]. In this study, non-linearity was assessed using a fractional polynomial non-linearity test.

### Multivariable Mendelian randomization

Multivariable Mendelian randomization (MVMR) is an extension of MR that allows for the causal effects of multiple exposures on the outcome to be estimated. MVMR estimates the “direct” causal effect of each exposure included in the estimate on the outcome, conditional on the other exposures included in the model. It is therefore particularly useful when two or more potentially related exposures are of interest and the researcher wishes to understand whether both exposures have a causal effect on the outcome [18].

### Statistical analysis

For statistical analysis in this study, R version 4.1.2 (R Foundation for Statistical Computing, Vienna, Austria) and SAS version 9.4 (SAS Institute Inc., Cary, NC, USA) were used. All statistical tests were two-sided, and a p-value of less than 0.05 was considered statistically significant.

### Ethics statement

This study protocol was reviewed and approved by the Institutional Review Board of Severance Hospital (Seoul, Korea), and informed consent was obtained (IRB No. 4-2011-0277).

## RESULTS

A total of 153,971 Korean participants with genetic information were included in the analysis. Baseline characteristics are presented in Table 1. The average follow-up period was 13 years, and there were 11,588 cases of ASCVD. Among those diagnosed with ASCVD, 34.0% were females, although males exhibited a higher incidence rate. The average age at onset was 53.8 years. The FSG level was approximately 11 mg/dL higher in the ASCVD event group compared to the non-event group. Additionally, body weight, waist circumference, SBP, LDL-cholesterol, and TG levels were all elevated in the ASCVD event group. Relative to the non-event group, the ASCVD event group had lower income levels, a higher smoking rate, and lower rates of physical activity and alcohol consumption.

Table 2 presents the hazard ratio of FSG levels for CVD, adjusted for sex, age, HDL-cholesterol, LDL-cholesterol, SBP, alcohol consumption, and smoking status. The data indicate an increased risk for all CVDs, with the exception of hemorrhagic stroke. Specifically, a 10 mg/dL increase in FSG level is associated with a 5% increase in the risk of ASCVD and stroke (hazard ratio [HR], 1.05; 95% confidence interval [CI], 1.04 to 1.06). The risk for thrombotic stroke rises by 6% (HR, 1.06; 95% CI, 1.05 to 1.08), for IHD by 5% (HR, 1.05; 95% CI, 1.04 to 1.07), and for myocardial infarction by 8% (HR, 1.08; 95% CI, 1.05 to 1.10).

Table 3 shows the OR for ASCVD associated with every 10 mg/dL increase in FSG, based on the crude 2-sample MR analysis. The analysis utilized FSG genetic variants from KoGES as the exposure and ASCVD data from KCPS-II as the outcome. In assessing the link with CVD, 42 FSG-related genetic variants were identified (Supplementary Material 2). Assuming uniformity in the instrumental variables, a significant association was found between FSG and ASCVD, IHD, and AMI. Specifically, the OR for a 10 mg/dL increase in FSG in relation to ASCVD was 1.11, which was statistically significant (95% CI, 1.04 to 1.18; p<0.001).

**Figure 2:**
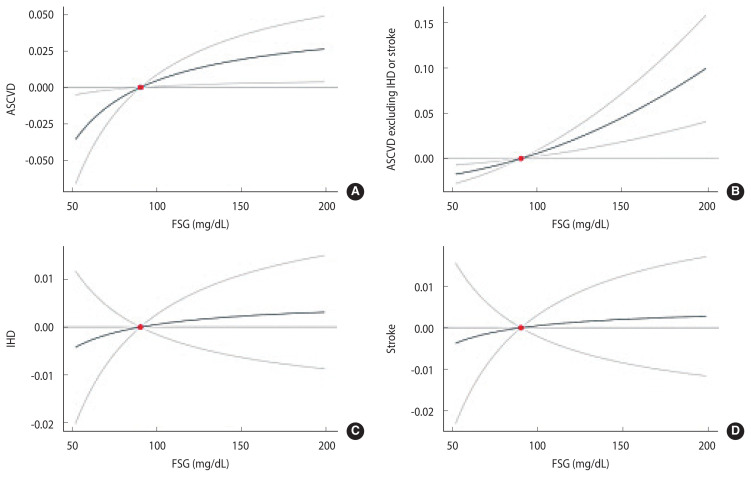
Non-linear Mendelian randomization (MR) between fasting serum glucose (FSG) and atherosclerotic cardiovascular disease (ASCVD) using fractional polynomial non-linearity. (A) ASCVD, (B) ASCVD excluding ischemic heart disease (IHD) or stroke, (C) IHD, and (D) stroke. The dotted line represents the 95% confidence interval.

Figure 2 shows the non-linear MR results for FSG and ASCVD. In the analysis, a linear relationship was observed between fasting blood sugar (FBS) and ASCVD, which includes IHD and stroke (Figure 2A and B). Although a non-linear relationship between FSG level and ASCVD is depicted in Figure 2A, the fractional polynomial non-linearity p-value was not significant (p=0.596). Similarly, in the subsequent Figure 2B (p=0.734), 2C (p=0.753), and 2D (p=0.659), no J-shaped curves were observed in relation to FBS levels; all demonstrated a linear relationship.

**Figure 3:**
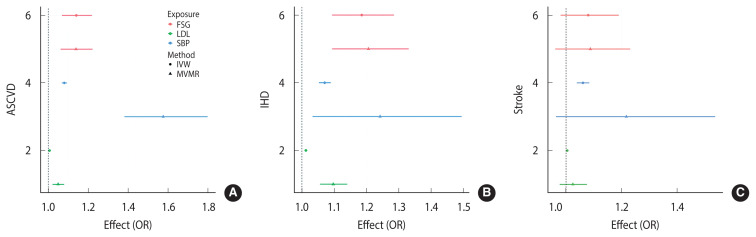
Multivariable Mendelian randomization (MVMR) analysis results for atherosclerotic cardiovascular disease (ASCVD). (A) ASCVD, (B) ischemic heart disease (IHD), and (C) stroke. FSG, fasting serum glucose; LDL, low- density lipoprotein; SBP, systolic blood pressure; IVM, inverse-variance weighted; OR, odds ratio.

Figure 3 displays the ORs for ASCVD, IHD, and stroke associated with every 10 mg/dL increase in FSG, based on both crude and multivariable MR analyses. This study utilized MVMR, employing KoGES data for FSG and KCPS-II data for ASCVD, IHD, and stroke. The F-value was used to assess the validity of the instrumental variables in the MVMR model. Additionally, the results of the crude MR were juxtaposed with those of the MVMR, using the IVW method for comparison. The variables included in the MVMR model were FSG levels of 10 mg/dL, SBP, and LDL-cholesterol.

The conditional F-statistics for instrument strength values of FSG, SBP, and LDL used in the MVMR model were 13.69, 7.41, and 38.50, respectively. The Q-statistic for instrument validity was 141.67 on 121 degrees of freedom (p=0.096). FSG showed a significantly positive causal relationship with ASCVD through crude MR (IVW circled) and MVMR analysis (marked with a triangle) (OR, 1.14; 95% CI, 10.7 to 1.21; Figure 3A). The Q-statistic for instrument validity was 142.30 on 121 degrees of freedom (p=0.090). FSG showed a significantly positive causal relationship with IHD through crude MR (IVW circled) and MVMR analysis (marked with a triangle) (Figure 2B). The Q-statistic for instrument validity was 134.33 on 121 degrees of freedom (p=0.192). FSG showed no significant causal relationship with stroke through crude MR (IVW circled) and MVMR analysis (marked with a triangle). Both SBP and LDL showed wide confidence intervals for MVMR and were not significant (Figure 2C).

## DISCUSSION

This study aimed to explore the causal relationship between FSG and the incidence of CVD in Koreans using MR research. Previous observational epidemiological studies have faced challenges, as their results could not be interpreted as causal due to confounding variables, measurement errors, and reverse causality. To address these issues, this study employed the MR approach, which utilizes genetic factors as instrumental variables [4,5]. Genetic data from KCPS-II and KoGES Biobanks were utilized for this purpose. The findings revealed that with every 10 mg/dL increase in FSG, the risk of ASCVD rose by 11%, IHD by 15%, and AMI by 37%. However, no significant associations were found concerning stroke events. These outcomes remained consistent in MVMR analyses that adjusted for genetic scores related to LDL-cholesterol and SBP.

In cohort studies, the impact of ASCVD per 10 mg/dL increase in FSG was less pronounced (HR, 1.05) compared to the effect observed in MR analysis (OR, 1.11). This discrepancy suggests that the true causal relationship may have been obscured by confounding factors or reverse causation in observational studies. To comprehensively elucidate the biological and molecular mechanisms linking hyperglycemia with the development of ASCVD, further experimental studies and larger prospective genetic studies are required.

### Previous studies

Although type 2 diabetes has long been recognized as a risk factor for coronary artery disease (CAD), the intricate interplay of various metabolic changes complicates the determination of whether the glycemic component influences CAD risk [19]. Epidemiological studies in this field are susceptible to confounding, and randomized controlled trials focusing on glycemic control have yielded inconclusive results. Genetic analyses employing a polygenic instrument within an MR framework can capture a significant proportion of variance in an endophenotype and establish causality for that trait.

Our findings corroborate previous studies indicating that elevated glucose levels significantly contribute to increased ASCVD risk. A clinical implication of our results is that they support the idea that reducing glucose levels may offer cardiovascular benefits, even for individuals without diabetes. Prospective epidemiological data consistently demonstrate that even minor changes in blood glucose can impact cardiovascular morbidity and mortality in healthy individuals [20]. An extended meta-regression analysis revealed a linear relationship, without a threshold effect, between FSG and CAD risk [2]. Furthermore, intensive glucose-lowering therapy decreased the risk of myocardial infarction by 15% to 20% in people with newly diagnosed type 2 diabetes [21] and in patients with established type 2 diabetes [22]. A meta-analysis of large randomized controlled trials showed that intensive glucose-lowering was associated with a significant 9% reduction in CAD events [23]. In an observational study by Park et al. [24], which monitored ASCVD according to FBS levels, a J-shaped relationship was observed between FBS and ASCVD. The J-shaped curve noted in Park et al. [24]’s study is considered a limitation of the observational study design. In our study, we did not observe any J-shaped relationship between FSG and ASCVD. This study suggests that lower blood sugar levels are associated with a reduced risk of ASCVD.

### Mechanisms of fasting serum glucose and atherosclerotic cardiovascular disease

Several mechanisms linking FSG and ASCVD have been reported in the literature. Here, we discuss some of the key mechanisms that connect FSG with ASCVD.

#### Insulin resistance

Elevated FSG levels are frequently linked to insulin resistance [25], a condition in which the body’s cells fail to respond adequately to insulin, a hormone that regulates blood sugar. This can result in increased FSG levels, subsequently elevating the risk factors for CVD, including hypertension and dyslipidemia [26].

#### Atherosclerosis

High FSG levels are associated with the development and progression of atherosclerosis, a condition characterized by the accumulation of fatty deposits in the arteries, which leads to reduced blood flow. Chronic hyperglycemia can damage the walls of blood vessels, thereby promoting atherosclerosis and elevating the risk of heart disease [27].

#### Inflammation

Elevated FSG levels can induce chronic low-grade inflammation throughout the body. This inflammation plays a crucial role in the development of atherosclerosis and can heighten the risk of cardiovascular events. High FSG levels are believed to contribute to this state of inflammation [28].

#### Dyslipidemia

Abnormal FSG levels can disrupt lipid metabolism, resulting in an unfavorable lipid profile characterized by elevated TG levels and reduced HDL-cholesterol levels. These lipid abnormalities can heighten the risk of CVD [29].

#### Oxidative stress

High blood sugar levels can exacerbate oxidative stress, which refers to an imbalance between the production of harmful free radicals and the body’s ability to counteract their effects. Oxidative stress can damage blood vessels and contribute to CVD [30].

#### Endothelial dysfunction

Chronic hyperglycemia can impair endothelial function, which is the inner lining of blood vessels. This dysfunction is a critical step in the development of atherosclerosis and can result in hypertension and decreased vascular flexibility, both of which are risk factors for CVD [31].

#### Platelet aggregation

Elevated blood sugar levels may promote abnormal platelet aggregation, increasing the risk of blood clots and thrombotic events. This can lead to heart attacks and strokes [32].

Individuals with stroke who also had diabetes exhibited pancreatic dysfunction (β-cell exhaustion), suggesting that hyperglycemia, rather than insulin resistance, is a risk factor for stroke [33]. Several key mechanisms contribute to the reduced bioavailability of endothelium-derived nitric oxide in diabetes. Hyperglycemia inhibits the production of nitric oxide by blocking the activation of endothelial nitric oxide synthase and by increasing the production of reactive oxygen species [34]. Acute hyperglycemia, induced by an intravenous glucose load in healthy individuals, led to an increase in QT interval and several sympathetic tone-dependent hemodynamic parameters, including a rise in blood pressure and elevated plasma concentrations of epinephrine and norepinephrine. These mechanisms support the idea that hyperglycemia alone can increase the risk of AMI in susceptible individuals by enhancing sympathetic tone and prolonging the QT interval [35].

### Strengths and limitations

The association between FSG and ASCVD was confirmed using data from two large-scale Korean Biobank studies. One of the strengths of our study is the reduction of concerns about confounding and reverse causation through the use of an MR approach. This method is advantageous because genetic variants, which are fixed at conception, are less likely to be associated with confounders compared to directly measured environmental exposures [36]. However, our findings should be interpreted with caution due to potential biases from other unmeasured factors that could affect the estimated impact of elevated FSG on ASCVD. In addressing these concerns, the MR method, particularly MVMR, was employed. Nonetheless, a limitation of this approach was the insufficient F-value of SBP as an instrumental variable. It is also acknowledged that non-genetic factors might have a significant influence on FSG and ASCVD risk. To address this, genetically determined FSG was analyzed. Future research should consider both genetic and environmental factors and their interactions. MR studies inherently assume conditions such as the absence of pleiotropy, no linkage disequilibrium confounding, consistent population structure, and strong genetic instruments [37]. Our analysis also presupposed a linear relationship between FSG and ASCVD. While FSG may exhibit a non-linear relationship with ASCVD, such patterns are typically observed in individuals with low glucose levels, who constitute a minority of the general population [38]. Lastly, it is important to note that our findings may not be generalizable to other ethnic groups, as our study was confined to Korean populations.

In conclusion, our findings support a causal relationship between increased FSG and the risk of ASCVD in the Korean population.

## Figures and Tables

**Figure f4-epih-46-e2024096:**
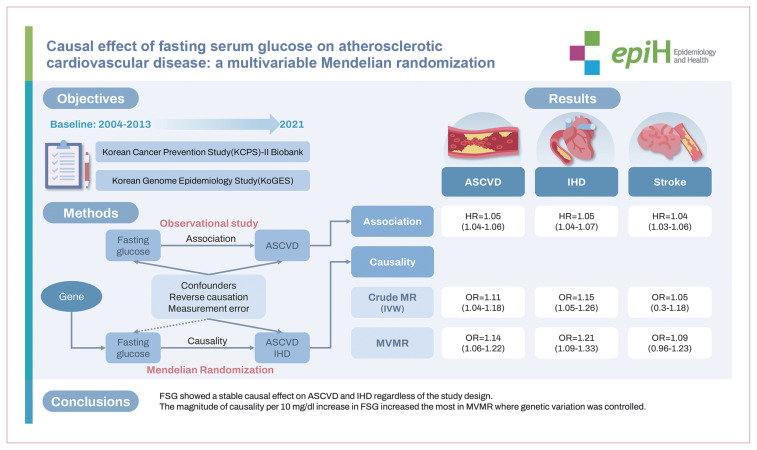


**Table 1:** Baseline characteristics of participants in the KCPS-II Biobank

Variables	ASCVD event (n=11,588)	No event (n=142,383)	p-value^1^	Age adjusted p-value
Female	34.0	40.0	<0.001	<0.001
Age and socioeconomic factors				
Age (yr)	52.9±11.4	40.8±10.0	<0.001	-
School education (yr)	12.7±3.8	14.4±3.0	<0.001	<0.001
Monthly income (10^4^ Korean won)	350±213	434±202	<0.001	<0.001
Lifestyle factors				
Ever smoking status	55.9	50.4	<0.001	<0.001
Alcohol drinking status	73.0	84.4	<0.001	0.148
Physical activity status	36.0	41.8	<0.001	0.201
Anthropometry and BP				
Fasting serum glucose (mg/dL)	101.0±30.1	90.2±17.5	<0.001	<0.001
Height (cm)	164.0±8.6	165.0±8.3	<0.001	<0.001
Weight (kg)	67.2±11.1	63.6±12.0	<0.001	<0.001
BMI (kg/m^2^)	24.8±3.1	23.2±3.2	<0.001	<0.001
Waist circumference (cm)	85.3±9.0	78.9±9.7	<0.001	<0.001
Systolic BP (mmHg)	126.0±15.8	116.0±14.4	<0.001	<0.001
Lipid profiles				
LDL-cholesterol (mg/dL)	114.0±34.3	111.0±31.0	<0.001	<0.001
HDL-cholesterol (mg/dL)	50.1±1.4	53.6±11.3	<0.001	<0.001
Triglycerides (mg/dL)	155±104	125±85	<0.001	<0.001
Self-reported disease				
HTN	53.9	15.0	<0.001	<0.001
DM	17.4	4.4	<0.001	<0.001

Values are presented as percentage or mean± standard deviation.

KCPS-II, Korean Cancer Prevention Study-II; ASCVD, atherosclerotic cardiovascular disease; BMI, body mass index; BP, blood pressure; LDL, low-density lipoprotein; HDL, high-density lipoprotein; HTN, hypertension; DM, diabetes mellitus.

1 Using independent t-test for categorical variables or chi-square test for continuous variables.

**Table 2:** Association with increased risk of cardiovascular events for every 10 mg/dL increase in fasting serum glucose using Cox proportional hazard anal model in observational study

Events	No. of event	Model 1^1^	p-value	Model 2^2^	p-value
Atherosclerotic cardiovascular disease	6,609	1.05 (1.04, 1.06)	<0.001	1.04 (1.03, 1.05)	<0.001
Total stroke	3,255	1.04 (1.03, 1.06)	<0.001	1.01 (0.99, 1.03)	0.331
Thrombotic stroke	1,451	1.06 (1.05, 1.08)	<0.001	1.03 (1.00,1.05)	0.062
Hemorrhagic stroke	633	1.03 (0.99, 1.06)	0.161	0.99 (0.93, 1.05)	0.374
Ischemic heart disease	4,478	1.05 (1.04, 1.07)	<0.001	1.04 (1.02, 1.05)	0.004
Acute myocardial infarction	775	1.08 (1.05, 1.10)	<0.001	0.99 (0.95, 1.04)	0.754

Values are presented as hazard ratio (95% confidence interval).

HDL, high-density lipoprotein; LDL, low-density lipoprotein.

1 Model 1: Adjusted for age, sex, HDL-cholesterol, LDL-cholesterol, systolic blood pressure, alcohol drinking status, and smoking status.

2 Model 2: Adjusted for age, sex, HDL-cholesterol, LDL-cholesterol, systolic blood pressure, alcohol drinking status, smoking status, body mass index, exercise, triglyceride, monthly income, and self-reported disease.

**Table 3:** Association with increased risk of cardiovascular events for every 10 mg/dL increase in fasting serum glucose using two-sample Mendelian randomization (MR)

Events	IVW	p-value	Weighted median	p-value	MR-Egger^1^	p-value	P for pleiotropy
ASCVD	1.11 (1.04, 1.18)	<0.001	1.07 (0.98, 1.16)	0.126	0.95 (0.79, 1.16)	0.666	0.111
Total stroke	1.05 (0.93, 1.18)	0.062	1.05 (0.91, 1.24)	0.153	0.76 (0.52, 1.09)	0.153	0.077
Thrombotic stroke	1.08 (0.89, 1.31)	0.411	1.02 (0.81, 1.31)	0.823	0.66 (0.37, 1.17)	0.168	0.087
Hemorrhagic stroke	1.18 (0.94, 1.48)	0.134	1.15 (0.84, 1.57)	0.365	0.86 (0.42, 1.75)	0.691	0.612
Ischemic heart disease	1.15 (1.05, 1.26)	0.001	1.12 (0.99, 1.28)	0.059	1.23 (0.92, 1.65)	0.162	0.643
Myocardial infarction	1.37 (1.03, 1.83)	0.033	1.28 (0.95, 1.74)	0.047	1.96 (0.79, 4.85)	0.152	0.419

Values are presented as odds ratio (95% confidence interval).

ASCVD, Atherosclerotic cardiovascular disease; IVW, inverse-variance weighted.

1 From MR-Egger intercept test, detection of directional pleiotropy.
