# Supplementary figures and images for: Causal effect of fasting serum glucose on atherosclerotic cardiovascular disease: a multivariable Mendelian randomization
Source: Epidemiol Health. 2024 Dec 6;46:e2024096. doi: 10.4178/epih.e2024096 (PMC11840400; doi:10.4178/epih.e2024096)

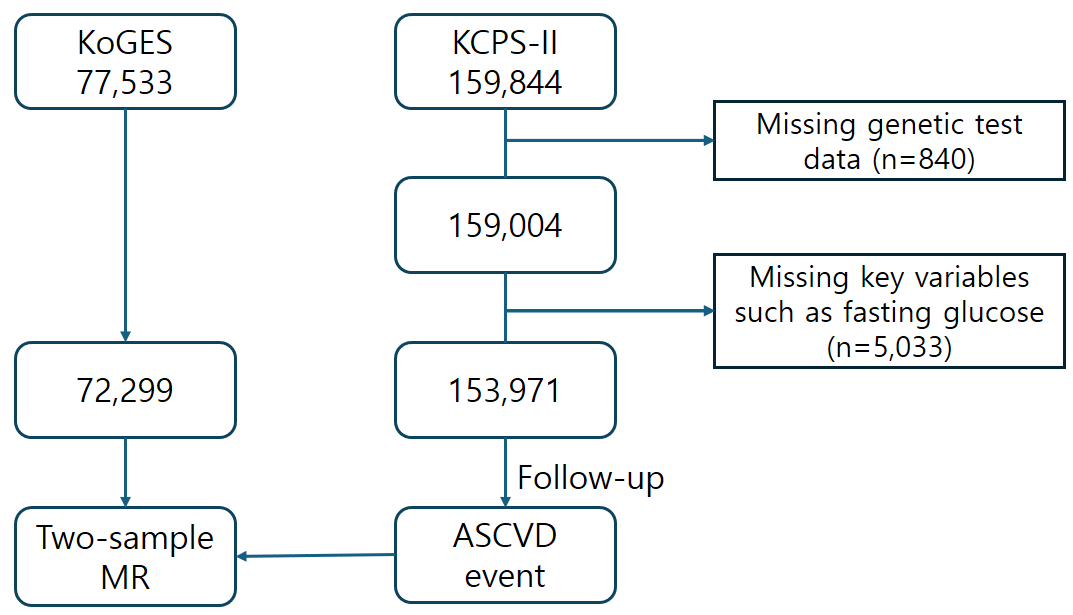


Supplementary Material 1. Flow chart for extracting research data.

Supplement: Supplementary file 1 [file epih-46-e2024096-Supplementary-1.docx]
