# Supplementary material for: Causal effect of fasting serum glucose on atherosclerotic cardiovascular disease: a multivariable Mendelian randomization
Source: Epidemiol Health. 2024 Dec 6;46:e2024096. doi: 10.4178/epih.e2024096 (PMC11840400; doi:10.4178/epih.e2024096)
Supplement: Supplementary file 2 [file epih-46-e2024096-Supplementary-2.docx]

Supplementary Material 2. A total of 42 genome-wide significant SNPs for FSG analyzed with KoGES data in the integrated 2SMR analysis of KoGES and KCPS-II data.

| no. | SNP | EA | OA | beta | se | p-value | EAF | palindromic | ambiguous |
| --- | --- | --- | --- | --- | --- | --- | --- | --- | --- |
| 1 | rs10281892 | G | A | 0.912077 | 0.105451 | 5.18E-18 | 0.4209 | FALSE | FALSE |
| 2 | rs10487796 | A | T | -1.04863 | 0.111372 | 4.71E-21 | 0.3225 | TRUE | FALSE |
| 3 | rs10811662 | A | G | -1.52069 | 0.105074 | 1.81E-47 | 0.4388 | FALSE | FALSE |
| 5 | rs10908278 | T | A | 0.761465 | 0.114807 | 3.30E-11 | 0.2911 | TRUE | FALSE |
| 6 | rs11065774 | A | G | -1.16649 | 0.140862 | 1.22E-16 | 0.1647 | FALSE | FALSE |
| 7 | rs11065983 | C | A | 0.689411 | 0.105895 | 7.50E-11 | 0.4299 | FALSE | FALSE |
| 8 | rs111366757 | A | T | 0.859267 | 0.135354 | 2.18E-10 | 0.1842 | TRUE | FALSE |
| 9 | rs111531379 | C | T | 2.24291 | 0.269681 | 9.03E-17 | 0.03875 | FALSE | FALSE |
| 11 | rs114173940 | T | A | 1.56127 | 0.282475 | 3.26E-08 | 0.03546 | TRUE | FALSE |
| 12 | rs11558471 | G | A | -1.12593 | 0.105741 | 1.78E-26 | 0.4177 | FALSE | FALSE |
| 13 | rs1260326 | C | T | 1.14811 | 0.105162 | 9.50E-28 | 0.4498 | FALSE | FALSE |
| 14 | rs12712928 | C | G | 1.08286 | 0.107503 | 7.28E-24 | 0.3745 | TRUE | FALSE |
| 16 | rs13229610 | T | G | -0.811456 | 0.106866 | 3.12E-14 | 0.3923 | FALSE | FALSE |
| 17 | rs1337919 | T | G | -0.981441 | 0.153764 | 1.74E-10 | 0.1328 | FALSE | FALSE |
| 18 | rs13387347 | C | T | 0.710883 | 0.104551 | 1.05E-11 | 0.4768 | FALSE | FALSE |
| 19 | rs1574285 | G | T | 0.835515 | 0.105313 | 2.13E-15 | 0.4246 | FALSE | FALSE |
| 20 | rs2241823 | C | A | 0.676988 | 0.104313 | 8.59E-11 | 0.4757 | FALSE | FALSE |
| 21 | rs2497351 | T | C | 1.65436 | 0.239911 | 5.36E-12 | 0.04959 | FALSE | FALSE |
| 22 | rs2525858 | A | G | -0.951821 | 0.148457 | 1.44E-10 | 0.1446 | FALSE | FALSE |
| 23 | rs35612982 | C | T | 1.49854 | 0.104153 | 6.17E-47 | 0.4658 | FALSE | FALSE |
| 24 | rs3859609 | T | C | -0.656165 | 0.106318 | 6.76E-10 | 0.3985 | FALSE | FALSE |
| no. | **SNP** | **EA** | **OA** | **beta** | **se** | **p-value** | **EAF** | **palindromic** | **ambiguous** |
| 25 | rs392794 | C | T | -0.607075 | 0.104367 | 6.00E-09 | 0.4835 | FALSE | FALSE |
| 26 | rs4258054 | C | T | -0.620922 | 0.110394 | 1.86E-08 | 0.3328 | FALSE | FALSE |
| 27 | rs4728092 | A | C | 0.963977 | 0.168086 | 9.75E-09 | 0.108 | FALSE | FALSE |
| 28 | rs56805921 | C | G | 0.633137 | 0.106961 | 3.23E-09 | 0.3934 | TRUE | FALSE |
| 29 | rs60415045 | C | A | 0.943069 | 0.109025 | 5.15E-18 | 0.3559 | FALSE | FALSE |
| 30 | rs60808706 | A | G | -1.33493 | 0.107036 | 1.06E-35 | 0.392 | FALSE | FALSE |
| 32 | rs635634 | T | C | 0.668475 | 0.11865 | 1.76E-08 | 0.2602 | FALSE | FALSE |
| 33 | rs6780171 | A | T | 0.660475 | 0.115097 | 9.56E-09 | 0.2906 | TRUE | FALSE |
| 34 | rs7129793 | T | C | -1.27221 | 0.218815 | 6.10E-09 | 0.06051 | FALSE | FALSE |
| 37 | rs7352806 | A | G | -0.598672 | 0.10457 | 1.03E-08 | 0.4641 | FALSE | FALSE |
| 38 | rs742761 | T | C | -0.71 | 0.12816 | 3.03E-08 | 0.212 | FALSE | FALSE |
| 39 | rs75214475 | C | T | -0.598825 | 0.107532 | 2.56E-08 | 0.384 | FALSE | FALSE |
| 40 | rs887688 | T | G | -0.738169 | 0.133615 | 3.30E-08 | 0.1878 | FALSE | FALSE |
| 41 | rs9350293 | A | C | 0.911404 | 0.107567 | 2.39E-17 | 0.3827 | FALSE | FALSE |
| 42 | rs9465773 | G | C | -0.727779 | 0.108642 | 2.10E-11 | 0.3654 | TRUE | FALSE |

EA, effect allele; OA, other allele; EAF, effect allele frequency
